# The Development and Evaluation of Melatonin-Loaded, Calcium Oxide Nanoparticle-Based Neem and Clove Extract: An In Vitro Study

**DOI:** 10.7759/cureus.46293

**Published:** 2023-09-30

**Authors:** Johnisha Harris, Sankari Malaiappan, Rajeshkumar S

**Affiliations:** 1 Periodontology, Saveetha Dental College and Hospitals, Saveetha Institute of Medical and Technical Sciences, Saveetha University, Chennai, IND; 2 Pharmacology, Saveetha Dental College and Hospitals, Saveetha Institute of Medical and Technical Sciences, Saveetha University, Chennai, IND

**Keywords:** green synthesis, calcium oxide nanoparticles, melatonin, clove, neem

## Abstract

Background

Various local drug delivery systems have been tried so far to target microorganisms responsible for periodontitis. However, none of them were effective enough to destroy the periodontal pathogens. This study aimed to analyze the antimicrobial, antioxidant, and anti-inflammatory properties of melatonin-loaded, calcium oxide nanoparticles-based neem and clove extract against oral pathogens to be further used as a local delivery agent.

Methodology

Powdered fresh neem leaves and clove buds were weighed, added to double distilled water, and then boiled for half an hour. Boiling helps in activating the phytochemicals present in the extract. The solution was boiled further to obtain a concentrated solution. To this 0.241 g of melatonin powder dissolved in 10 mL of double distilled water was added to the previous mixture and left undisturbed in a stirrer overnight.

Results

The properties of the extract such as antioxidant, antibacterial, anti-inflammatory, cytotoxicity, and embryonic toxicology were studied. In the case of antimicrobial activity, at 100 μg/mL, the zone of inhibition (ZOI) was the highest at 18 ± 0.16 μg/mL and the lowest at 13 ± 0.3 at 25 μg/mL for *Candida albicans*. Similarly, at 100 μg/mL, the ZOI was the highest at 15 ± 0.25 μg/mL and the lowest was 13 ± 0.12 at 25 μg/mL for *Streptococcus mutans* and *Staphylococcus aureus*. Similarly, in the case of antioxidant and anti-inflammatory properties, they showed increased activity with increased concentrations of 10, 20, 30, 40, and 50 μg/mL.

Conclusions

This study proves that melatonin-added extracts have antimicrobial, antioxidant, anti-inflammatory, and cytotoxic properties which are almost equal to that of the standard. This indicates that they can be possibly further used as local delivery drugs. Further animal or cell line studies should be conducted before experimenting this is in clinical trials for periodontitis patients.

## Introduction

Periodontitis is an inflammatory disorder that is characterized by gingival bleeding, the development of periodontal pockets, and the degeneration of connective tissue attachment. The immune system is stimulated against bacteria present in the tooth biofilm, where the disease first manifests itself. Plaque-induced gingival inflammation is the most known form of periodontal disease in people and can lead to more severe forms of the condition. Gingival tissue and alveolar bone are severely lost in the advanced stage of the disease.

During the pathogenesis of this disease, free radicals are produced from the bacteria as well as inflammation and this creates a triggered immune response, which is a significant factor in periodontal disease. This event leads to the pro-inflammatory molecules getting activated and further causes periodontal destruction [1]. It also results in a massive increase in the nitrogen and oxygen reactive species which leads to oxidative tissue damage. The free radicals increase as the defense provided by the antioxidant properties is reduced due to the inflammation and this imbalance may lead to a considerable detrimental impact on the periodontal tissues [2]. This symbolizes that the drugs that are used against periodontitis should contain the potential to act against the pathogens. The potential therapeutic effects of green synthesized drugs in periodontitis have gained attention in the past few years because of their fewer side effects.

Green synthesis, the environmentally friendly synthesis or sustainable synthesis, refers to the development of processes and methodologies that minimizes or avoids the use of harmful substances and reduce the production of waste [3]. This was validated by another study by Pal et al. that green synthesis has gained significant attention in various fields, including pharmacology for the synthesis of herbal-based drugs to overcome the adverse effects of chemical drugs. When it comes to the green synthesis of drugs, researchers focus on finding alternative, environmentally friendly routes to produce pharmaceutical compounds. Among the various plants available, neem and clove were chosen considering their medicinal properties. Neem (*Azadirachta indica*) is a tree originating from the Indian subcontinent and is known for its numerous medicinal properties. Various parts of the neem tree, including the leaves, bark, seeds, and oil, have been traditionally used in Ayurvedic medicine for centuries [3]. Clove (*Syzygium aromaticum*) is a spice native to Indonesia and is widely used in medicine. Cloves are the dried flower buds of the clove tree and possess several medicinal effects due to their active compounds. Both of these are well known for their strong antimicrobial properties, particularly against bacteria and fungi. They can help inhibit the growth of various pathogens. They contain anti-inflammatory compounds that can help subside the inflammation [4]. They also have antioxidant properties that protect cells from oxidative damage, thereby neutralizing the reactive species. However, these properties do not seem to be strong enough and sufficient against periodontal pathogens without a powerful additive.

One such compound, which is well known for its activity against periodontal pathogens is melatonin. Permuy et al. conducted an extensive review of melatonin which is a derivative of the amino acid tryptophan. Chemically, melatonin is known as N-acetyl-5-methoxytryptamine. It has been studied for its potential applications in dentistry, particularly in the context of its various properties. It has also been found to influence bone metabolism by promoting bone formation and inhibiting bone resorption. This aspect is particularly relevant in periodontics, as the diseases can lead to bone loss around the teeth. The effects of melatonin on bone may help support periodontal tissue regeneration [5]. To effectively deliver these substances, a suitable vehicle is mandatory. Using nanoparticles as a potential drug delivery system due to their unique properties has been a focus for the past few years by authors like Vaseenon et al. Especially among them, calcium oxide (CaO) nanoparticles are generally considered to be biocompatible and allow them to penetrate biological barriers and reach target sites more effectively. The nanoparticles can be engineered to have a controlled size and surface characteristics, enabling improved drug loading and release profiles. In our study, we synthesized calcium oxide nanoparticles (CaONPs) using neem and clove extract without the use of any other hazardous chemicals or synthetic agents [6]. The study aims to analyze the antimicrobial, anti-inflammatory, antioxidant, cytotoxicity, and embryonic toxicology properties of melatonin-loaded neem and clove extract.

## Materials and methods

Synthesis of extract

Neem leaves and clove buds were the primary ingredients. They were collected, cleaned, and dried for almost five days to remove moisture. After thorough drying, the dried neem leaves as well as clove buds were crushed and made into a powder form. Accurately, 1 g of each powder was taken and then added to 100 mL of double distilled water. It was then boiled at 70°C for around 30 minutes. The boiling helps in activating the phytochemicals present in the extract. The solution was boiled further to obtain a concentrated solution [7].

Biosynthesis of CaONPs

The process began by mixing 10 mL of the extract and 90 mL of the CaO solution. An optimum ratio of 9:1 was maintained. Then, the prepared mixture was placed in a stirrer for 48 hours at optimal room temperature. The visual color change from dark yellow to light yellow denotes the formation of CaONPs. At 10,000 rpm, after centrifugation for about five minutes, the pellets were collected. They were later washed in double-standard purified water, then again with ethyl alcohol to obtain maximum purity. The obtained nanoparticle-containing powder was collected through lyophilization. The final product was stored at room temperature.

Preparation of melatonin mixture

To prepare this mixture, 0.241 g of melatonin powder was dissolved in 10 mL of double distilled water. This was uniformly mixed with the help of a vortex mixer. Later, this mixture was added to the prepared neem and clove extract and left in a stirrer overnight. Then, the final mixture was tested for various properties.

Anti-inflammatory activity

Egg Albumin Denaturation Assay

To perform egg albumin denaturation assay, 0.2 mL of fresh egg albumin and 2.8 mL of phosphate buffer were mixed. Different concentrations (10-50 µg/mL) of melatonin-loaded CaONPs extract were added to the mixture. The pH was adjusted to 6.3. Then, it was kept for 10 minutes at room temperature. Incubation was done in a water bath at 55°C for half an hour. The standard group contained diclofenac sodium while dimethyl sulphoxide was used as a control. Then, the samples were measured spectrophotometrically at 660 nm [8]. The percentage of protein denaturation was determined utilizing the following equation: % inhibition = (absorbance of control - absorbance of sample)/absorbance of control × 100.

Membrane Stabilization Assay

In vitro membrane stabilization assay is a widely used technique for evaluating the membrane stabilizing properties of natural and synthetic compounds. This assay analyzes the potential of a compound to stabilize the cell membrane by preventing its disruption and subsequent release of intracellular contents. The materials included tris-HCl buffer, human red blood cells (RBCs), phosphate-buffered saline (PBS), different concentrations of melatonin-loaded CaONP extract (10-50 µg/mL), centrifuge tube, and UV-visible spectrophotometer.

Preparation of RBC Suspension

Fresh human blood was collected in a sterile tube containing anticoagulants. The blood was centrifuged at 1,000 g for 10 minutes at optimal room temperature to separate the RBCs from other blood components. The supernatant was removed and washed the RBCs three times with PBS. Resuspend the RBCs in the tris-HCl buffer to obtain a 10% (v/v) RBC suspension.

Assay Procedure

Overall, 1 mL of the RBC suspension was pipetted into each centrifuge tube. Then, different concentrations of melatonin-loaded CaONP extract were added to each tube. The tubes were mixed gently and incubated at 37°C for half an hour. Subsequently, the tubes were centrifuges at 1,000 rpm for 10 minutes at room temperature to pellet the RBCs. At a wavelength of 540 nm, the absorbance of the supernatant was calculated with the help of a UV-visible spectrophotometer. The percentage inhibition of hemolysis was calculated using the following formula: % inhibition = (OD control - OD sample)/OD control) × 100.

OD control is the absorbance of the RBC suspension without the test compound(s) and OD sample is the absorbance of the RBC suspension with the test compound.

Antioxidant activity of CaONPs

Hydrogen Peroxide Assay

Overall, 1 mL of reaction mixture with 100 mL of 28 mM of 2-deoxy-2-ribose was prepared. To that various concentrations of Andrographis paniculata-mediated silver nanoparticles (10-50 μg/mL) were added. Along with that, 200 mL of ferric chloride, 200 µL of ethylenediaminetetraacetic acid, and 100 mL of ascorbic acid were mixed. Then, it was incubated for an hour at 37°C and the OD was measured at the wavelength of 532 nm against the blank solution. Tocopherol was chosen as a control. The following formula was used: hydroxyl radical scavenging activity (%) = ((A blank - A sample)/Ablank) × 100, where A blank is the absorbance of the control reaction (without sample), and A sample is the absorbance of the reaction with the sample.

Ferric-Reducing Antioxidant Powder Assay

Reagents used: (a) 300 mM acetate buffer, pH 3.6: 3.1 g of sodium acetate trihydrate was weighed, 16 mL of glacial acetic acid was added, and then 1 L of distilled water was added to make the volume. (b) TPTZ (10 mM in 40 mM HCl; M.W. 312.34), 2, 4, 6-tripyridyl-s-triazine. (c) FeCl_3_. 6 H_2_O (20 mM; M.W. 270.30). Just before testing, the functioning ferric-reducing antioxidant powder (FRAP) reagent was made by combining components a, b, c in ae ratio 10:1:1. FeSO_4_. 7H_2_O: 0.1 to 1.5 mM in methanol served as the standard. The Merck (Germany) firm prepared all regents.

Procedure: Distilled water (0.4 mL) and FRAP solution (3.6 mL) were combined and incubated at 37°C for five minutes. Then, this solution was combined with an 80 mL concentration of plant extract, which was then incubated for 10 minutes at 37°C. At 593 nm, the absorbance was gauged. The values were determined as for sample solutions, and five concentrations of FeSO_4_, 7H_2_O (0.1, 0.4, 0.8, 1, 1.12, and 1.5 mM) were used to generate the calibration curve.

Antimicrobial activity

Using the agar well diffusion method, the antibacterial activity of melatonin-loaded CaONP extract was assessed. We prepared and sterilized Mueller-Hinton agar plates in an autoclave at 121°C for 15-20 minutes. The medium was applied to the sterile petri plate surface after sterilization and allowed to cool to room temperature. Using sterile cotton swabs, the bacterial suspension (*Streptococcus mutans*, *Lactobacillus *spp., *Staphylococcus aureus*, and *Candida albicans*) was equally distributed across the agar plates. The agar plates were divided into 9 mm-diameter wells using a sterile polystyrene tip. Then, melatonin-loaded CaONP extracts at varying quantities (25 g, 50 g, and 100 g) were added to the wells. The standard was an antibiotic, such as bacteria-amoxyrite or fungi-flucanazole. For fungi cultures, plates were incubated at the temperature of 37°C for one and two days. By measuring the diameter of the inhibitory zone surrounding the wells, the antibacterial activity was assessed. A ruler was used to measure the diameter of the zone of inhibition (ZOI), record it in mm, and compute the ZOI.

Cytotoxic effect

Brine Shrimp Lethality Assay

Overall, 2 g of salt which does not contain iodine was mixed in 100 mL of double distilled water. In six well enzyme-linked immunosorbent assay plates, 11-12 mL volume saline was added. Then, 10 nauplii were placed in each well. The nanoparticles were added in various concentrations. The plates were kept for incubation for 24 hours. After 24 hours, the count of live nauplii was determined using the following formula: number of dead nauplii/number of dead nauplii + number of live nauplii × 100.

Zebrafish embryonic toxicology evaluation of CaONPs

Fish Maintenance Protocol and Exposure to CaONPs

Wild-type zebrafish were kept in separate tanks under controlled conditions of temperature (280 ± 20°C). Twice a day, optimum food was fed to the zebrafishes. Zebrafish embryos were obtained from each breeding unit, and viable eggs were collected and washed thoroughly thrice with a freshly prepared medium which is free of methylene blue. The study involved the placement of fertilized eggs in culture plates of varying well sizes (six, 12, and 24 wells) with 20 embryos per 2 mL solution per well. The experimental treatment and control groups were replicated three times. To prepare the experimental treatment, a stock suspension of melatonin-loaded CaONP extract with five different concentrations was freshly made and added directly to the E3 medium. To disperse the nanoparticles, the solution was placed in a sonicator for 15 minutes. A pH range of 7.2-7.3 was maintained. Nourished unaffected ones were treated with four specific concentrations of CaONPs for 24 to 96 hours post-fertilization. The CaONPs were added to the E3 medium in which the embryos were incubated. Control groups are the untreated ones. Every 12 hours, dead embryos were disposed of. The experimental plates should be covered in aluminum foil to eliminate light exposure. They were maintained at an optimal temperature of 28°C.

Evaluation of Zebrafish and Its Developmental Stages

Following fertilization, throughout the exposure period, the growth of zebrafish embryos was monitored using a high-definition microscope. The embryos were treated with different concentrations of our extract (5, 10, 20, 40, and 80 µg/L) for 24-78 hpf. Embryonic mortality as well as hatching rates were assessed at 24-hour intervals. The study parameters included embryo/hatchling mortality, hatching rate, and the identification and documentation of any malformations among them in control and treatment groups. Photographic documentation of affected embryos was captured using a COSLAB-light microscope, and the percentage of affected embryos was recorded every 24 hours.

## Results

Anti-inflammatory activity

**Figure 1:**
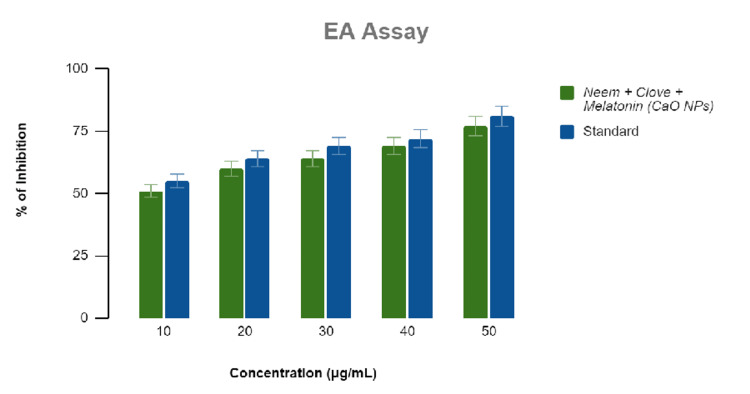
Graphical representation of the anti-inflammatory activity of melatonin-loaded neem and clove extract using egg albumin (EA) assay.

**Figure 2:**
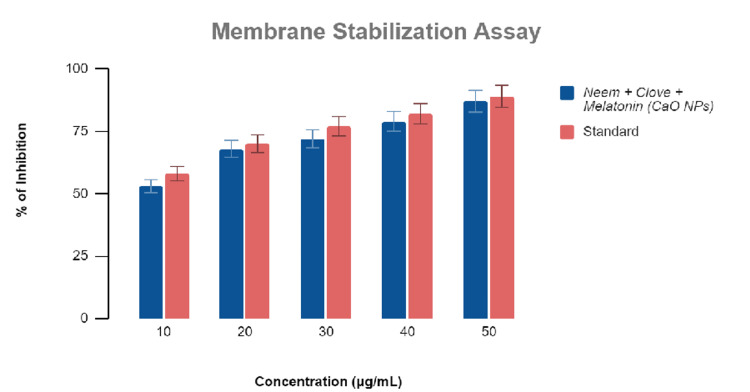
Graphical representation of the anti-inflammatory activity of melatonin-loaded neem and clove extract using membrane stabilization assay.

Melatonin-loaded CaONP-based neem and clove extract showed an increase in activity with increasing concentrations of 10, 20, 30, 40, and 50 μg/mL extract against similar concentrations of standard diclofenac sodium. The following calculations were done based on the reading that was obtained (Figures 1, 2). The activity was tested using three different assays, namely, egg albumin assay and membrane stabilization assay, respectively, to get an unbiased result.

Antioxidant activity

**Figure 3:**
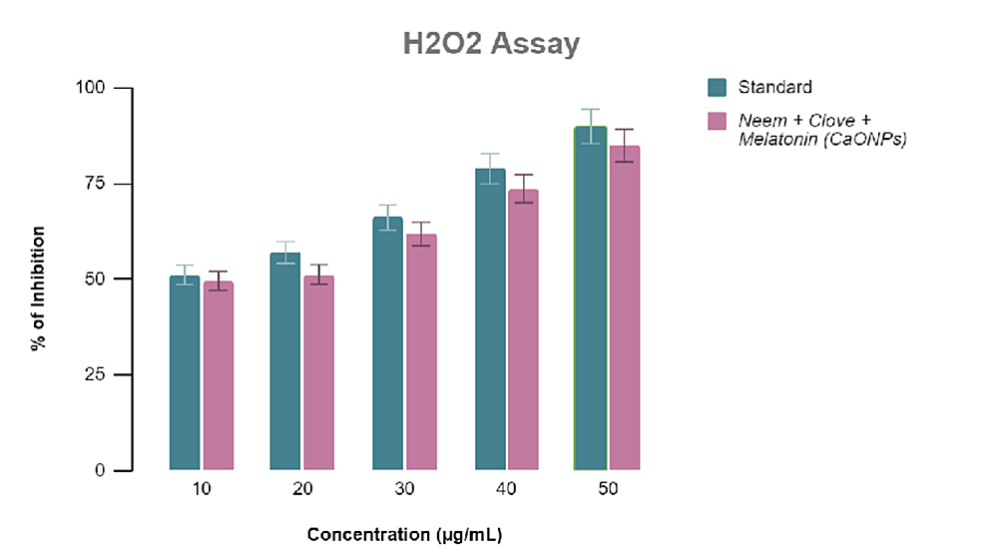
Graphical representation of the antioxidant activity of melatonin-loaded neem and clove extract using the hydrogen peroxide assay.

**Figure 4:**
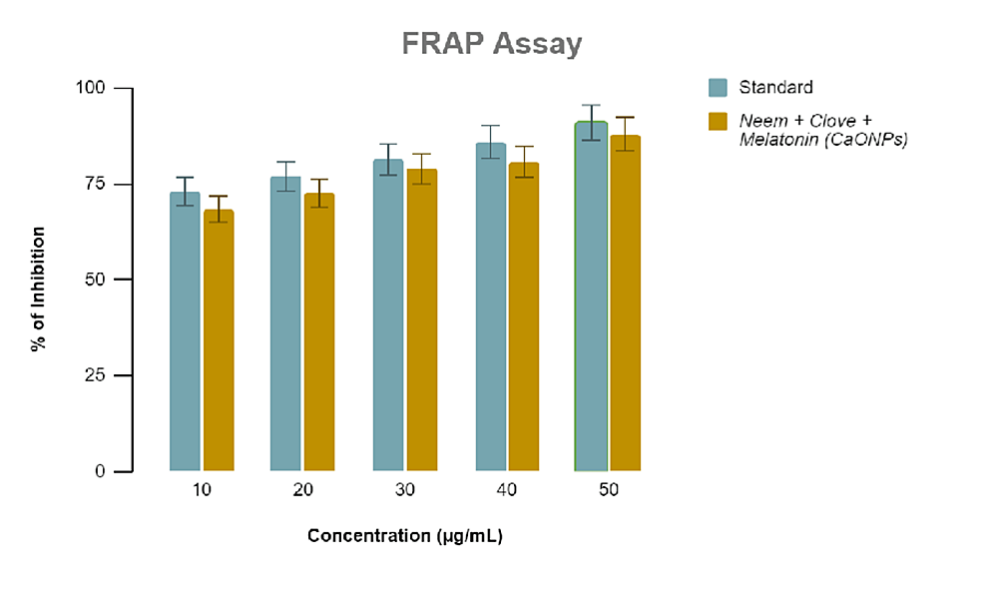
Graphical representation of the antioxidant activity of melatonin-loaded neem and clove extract using ferric-reducing antioxidant powder (FRAP) assay.

Antioxidant activity was determined using the hydrogen peroxide and FRAP assays. In past research, CaONPs showed good antioxidant activity. Therefore, it is evident that the additional antioxidant activity can be attributed to the melatonin present in the extract. Similar results were obtained from all the assays, which signifies the reliability of the results. According to Figure 3 and Figure 4, antioxidant activity increases with increase in concentrations of the extract. The difference in the antioxidant activity between the standard ascorbic acid and the melatonin-loaded CaONP-based neem and clove extract is only <5% in terms of ZOI, as validated by hydrogen peroxide and FRAP assays.

Antimicrobial activity

**Figure 5:**
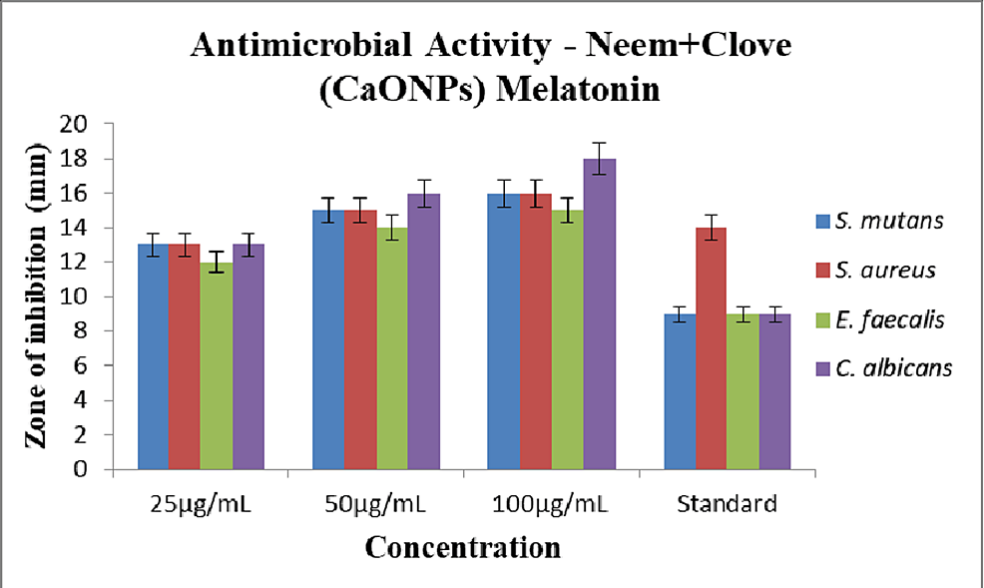
Graphical representation of the antimicrobial activity of melatonin-loaded neem and clove extract.

The antimicrobial activity of melatonin-loaded CaONP-based neem and clove extract was demonstrated using bacterial cultures by means of ZOI. Figure 5 represents the increase in ZOI with increased concentration of the melatonin extract. Hence, at 100 μg/mL, the ZOI was highest with 18 ± 0.16 and lowest was 13 ± 0.3 at 25 μg/mL for *C. albicans*. These results indicate that the extract was almost twofold greater than the standard. Similarly at 100 μg/mL, the ZOI was highest at 15 ± 0.25 and lowest was 13 ± 0.12 at 25 μg/mL for *S. mutans* and *S. aureus*. Whereas for *Enterococcus faecalis*, the highest activity was seen at the maximum concentration with the ZOI of 15 ± 0.23 μg/mL and the least at the lowest concentration with the ZOI of 12 ± 0.32 μg/mL (Table 1). This can also be attributed to the CaONPs used which provide a high surface area . This allows them to effortlessly come in contact with cell membrane causing damage to the genetic material which eventually leads to death. This death might be due to endocytosis or direct diffusion [9].

**Table 1:** The zone of inhibition around S. mutans, S. aureus, E. faecalis, and C. albicans on treatment with melatonin-loaded neem and clove extract under various concentrations of 25, 50, 100 µg/mL and standard diclofenac sodium.

Organism	25 µg/mL	50 µg/mL	100 µg/mL	Standard
S. mutans	13	15	16	9
S. aureus	13	15	16	14
E. faecalis	12	14	15	9
C. albicans	13	16	18	9

Cytotoxic effect

**Figure 6:**
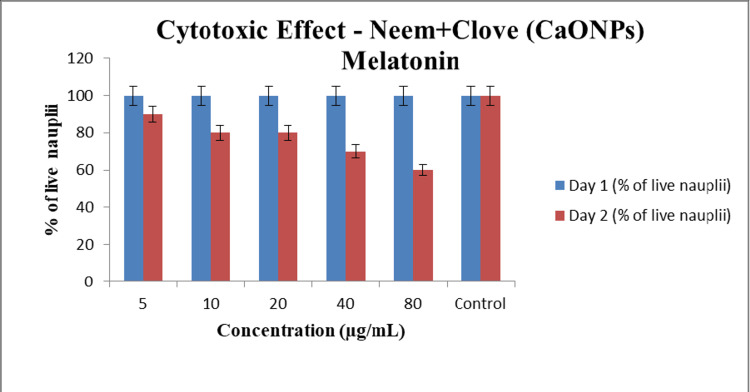
Graphical representation of cytotoxicity of melatonin-loaded neem and clove extract using brine shrimp.

A total of 60 live nauplii were taken and distributed into 10 nauplii each into each well. The effect of the melatonin-loaded CaONP-based neem and clove extract was observed in an interval of 24 hours [10]. It was observed that on the first day all 60 nauplii were alive at various concentrations of 5, 10, 20, 40, and 80 μL (Figure 6). However, on the second day, only 54 nauplii were alive in the first well of the microplate with a concentration of 5 μL, 48 nauplii were alive in the second well with a concentration of 10 μL, 48 nauplii were alive in the third well with a concentration of 20 μL, 42 nauplii were alive in the third well with a concentration of 40 μL, and 36 nauplii was alive in the third well with a concentration of 80 μL (Figure 6).

Embryonic toxicology

**Figure 7:**
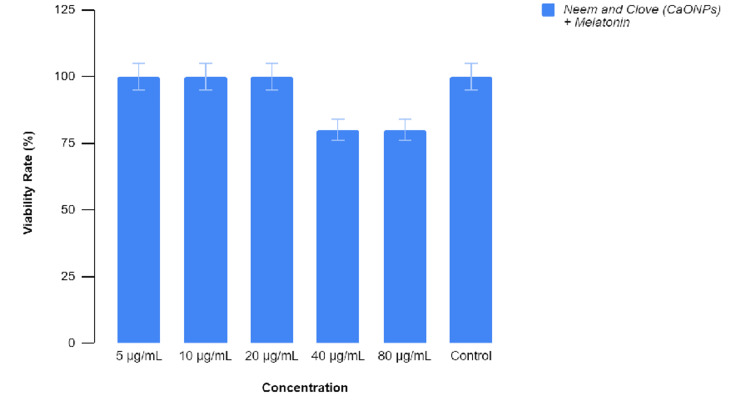
Graphical representation of the viability rate of wild zebrafish on treatment with melatonin-loaded neem and clove extract.

**Figure 8:**
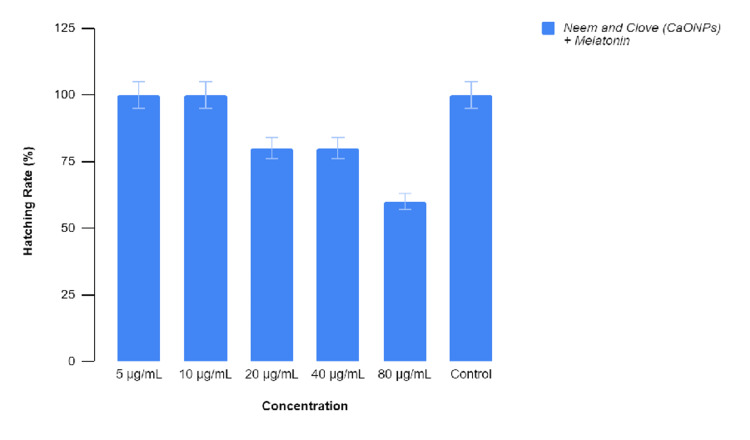
Graphical representation of the hatching rate of wild zebrafish on treatment with melatonin-loaded neem and clove extract.

**Figure 9:**
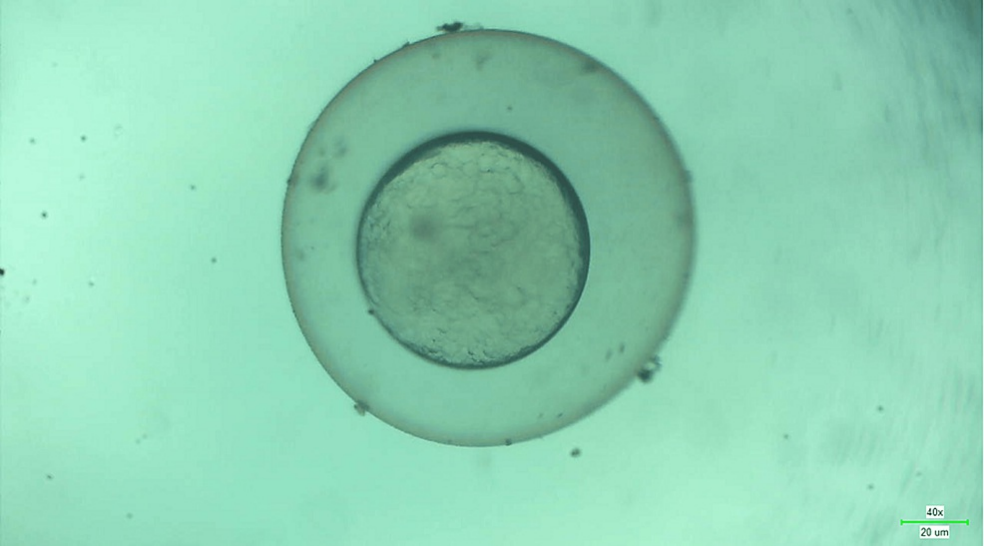
Microscopic image of wild zebrafish at 24 hpf with no signs of toxicity when treated with 80 µg/mL of melatonin-loaded neem and clove extract.

**Figure 10:**
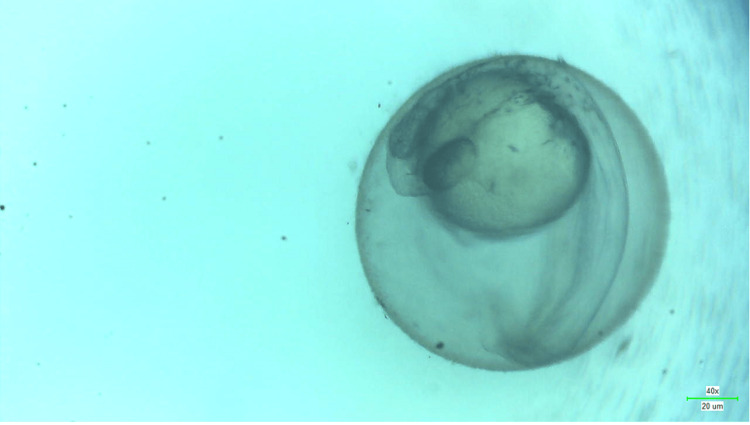
Microscopic image of wild zebrafish at 48 hpf showing signs of yolk sac edema when treated with 80 μg/mL of melatonin-loaded neem and clove extract.

**Figure 11:**
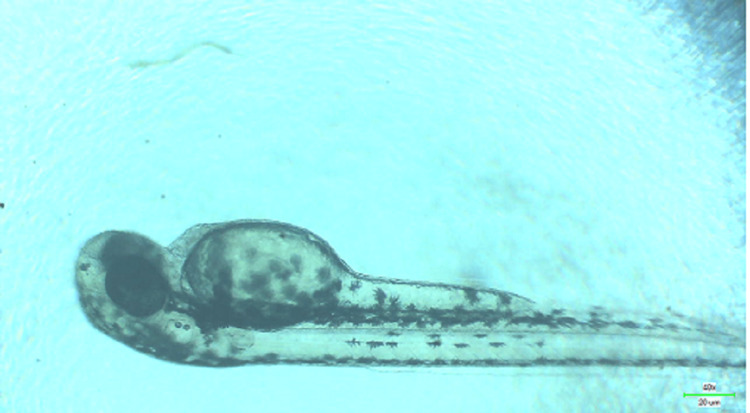
Microscopic image of wild zebrafish at 96 hpf showing signs of pericardial edema when treated with 80 µg/mL of melatonin-loaded neem and clove extract.

The embryos were treated with various concentrations of the prepared extract before 4 hpf, which is the sphere stage, and their developmental abnormalities were observed. Figure 7 represents the toxicity of the test and control groups which are untreated and treated groups, respectively. There were no significant (p > 0.05) changes during the exposure at the concentrations of 5, 10, and 20 μg/mL. However, on exposure to higher concentrations of 40 and 80 μg/mL of the extract, there was highly significant effect (p < 0.01) on embryo’s mortality rate. This significant rise in the mortality rates was observed up to 96 hpf on increasing the concentration of melatonin-containing extract. At the concentration of 80 μg/mL, there was a mortality rate of 25% at the stage of 96 hpf [11]. Figures 8-11 show the developmental stages of wild zebrafish at 24, 48, and 96 hpf at the maximum concentration of 80 μL.

Similarly, the hatching rate of zebrafish embryos were also affected depending on the concentrations. There was hatching rate of 100% in untreated embryos. However, at the concentrations of 20 and 40 μg/mL of the extract, embryos showed a 75% hatching rate. There were no significant differences between two concentrations. Their p-value was less than 0.01. Whereas up to a concentration of 80 μg/mL, the hatching rate was moderately significant (p < 0.05). Figure 8 represents the significant delay in the hatching ability as well as retardation of growth with developmental toxicity on increasing the concentrations of the extract. There was no problem with the zebrafish growth in the untreated group. In lower concentrations such as 5 and 10 μg/mL, there were no significant malfunctions up to the 96 hpf stage. Above 10 µg/mL, there was flexure as well as the truncation of the tail and spinal cord, edema in the yolk sac, and abnormalities of the fin. On microscopic examination, axial and tail bend along with pericardial edema wax observed. This was indicated by hypoplasia of head and eye, shrunken digestive gut and absence of swim bladder in the treated embryos. The abnormality was evident in the concentration of 80 μg/mL at almost all stages of embryo 24, 48, and 96 hpf.

## Discussion

Melatonin, which is well known for its vital role in regulating the circadian rhythm, has been widely studied for its potential effects on various physiological processes. While the primary use of melatonin is related to sleep disorders and jet lag, it has also been explored in other areas of healthcare, including dentistry, especially periodontics. In the field of periodontics, melatonin has attracted attention due to its antioxidant and anti-inflammatory properties. Chronic periodontitis is an inflammatory condition that affects the supporting tissues of the teeth, including the periodontal ligament and bone. Inflammation plays a vital role in the progression of periodontal disease, and reducing inflammation is an essential aspect of its management. Research suggests that melatonin may help in the treatment of periodontal disease through its anti-inflammatory effects. Additionally, melatonin’s antioxidant properties may contribute to its potential benefits in periodontics [12].

Melatonin’s ability to reduce free radicals and reduce oxidative stress may help protect periodontal tissues from damage. While the research on melatonin in periodontics is still limited, preliminary studies and experimental data have shown promising results. However, more comprehensive clinical trials are needed to establish its efficacy and determine the optimal dosage, delivery methods, and treatment protocols. Rana et al., in their study about melatonin against metal toxicity, reported that gram-negative organisms have a double membrane layer that contains a variety of lipids, which produces a permeability barrier that prevents the entry of some drugs [12]. Melatonin can easily penetrate cell membranes due to its higher lipophilic nature. *Porphyromonas gingivalis* uses heme to produce metabolic energy by degrading hemoglobin with the aid of the proteinase-adhesin complex. Melatonin, however, binds to iron, copper, and zinc due to its affinity for metal ions, making it unavailable for bacterial sustenance.

According to the data by Drobnik et al., the melatonin-induced increase in collagen content in the infected area that was seen in vivo may be caused by the direct action of melatonin on the fibroblasts. Furthermore, the activation of melatonin membrane receptors on collagen-producing cells determines whether this impact occurs [13]. Cutando et al. demonstrated that diabetes patients with periodontitis who applied 1% melatonin orabase cream topically to their gingiva could reduce their probing pocket depth and gingival index. Additionally, the use of 1% melatonin cream resulted in a large increase in salivary osteoprotegerin and a significant drop in salivary RANKL [14]. The study conducted by Ganganna et al. revealed that even after a lengthy incubation period of 72 hours, the minimal inhibitory concentration (MIC) value against *Aggregatibacter actinomycetemcomitans* (6.25 g/mL after 24 hours) remained constant. At the end of 72 hours, the MIC for *P. gingivalis* and *Fusobacterium nucleatum* increased [15]. Up to a concentration of 1 mM MEL and 5-MTX were proven to be biocompatible for human fibroblasts.

The adverse effect found because of long usage was drowsiness after oral intake [16]. In addition to its potential against reactive oxygen species and advantageous effects on bone metabolism, MEL also increases the expression of certain collagenous, non-collagenous proteins and pro-inflammatory mediators such as IL-10, thereby lowering the matrix metalloproteinases and tissue inhibitors of metalloproteinases ratio, and promotes healing by primary intention, all of which point to the possibility that it may help preserve and restore the integrity of gingival tissues [17]. Similarly, melatonin-loaded CaONP-based neem and clove extracts have also shown increased antioxidant activity as validated by hydrogen peroxide and FRAP assay in comparison with ascorbic acid as control. Balaji et al. suggested that melatonin is a potent local deliverable drug with minimal side effects [18]. Meenakshi et al. reported that melatonin levels were found to be higher in patients with healthy gingiva compared to those with periodontitis [19]. A clinical study conducted by Pawane et al. proved that local administration of melatonin improved the clinical parameters significantly when compared to those without local intervention [20].

This study supports the results of our study which shows an increase in the antimicrobial activity with increasing concentration of melatonin-loaded CaONP-based neem clove extract. According to Wang et al., melatonin reduces the expression of pro-inflammatory cytokines and boosts the levels of antioxidants, thereby reducing the oxidative stress associated with periodontal inflammation [21]. Similarly while exploring the properties of neem in periodontitis, Elavarasu et al. concluded that neem possesses antimicrobial properties using the Mueller-Hinton agar plates method with ZOI ranging from 29-34 mm under various concentrations against oral pathogens [22]. The gingival problems which manifest as minor symptoms such as bleeding or swollen gums later develop into tooth mobility or purulent exudates [23]. These problems can be influenced by various risk factors which might aggravate the progression of the disease [24]. Hence, proper knowledge of these influences on the molecular level might help to develop periodontal vaccines with agents similar to melatonin which can prevent the disease [25].

Limitations

There is very limited literature evidence for clove, especially in the treatment of periodontitis. Hence, this study might provide insights into the effects of *Syzygium aromaticum* on the periodontal tissues during intervention. Further, an antimicrobial assay against periodontal pathogens should be done to test its application as a local delivery drug in periodontitis.

## Conclusions

We have successfully biosynthesized melatonin-loaded CaONPs using neem and clove oil extract, which is the first study to try this combination of herbs. The phytochemicals included in the extract play the role of both reducing and helping in stabilizing the extract, limiting the usage of hazardous chemicals in the synthesis process. As the phytochemicals of the herbs were used as stabilizing agents, the use of toxic chemicals was avoided. The properties of the extract such as antioxidant, antibacterial, anti-inflammatory, cytotoxicity, and embryonic toxicology were studied. All properties showed minimal differences when compared to the standard in terms of anti-inflammatory, antioxidant, antimicrobial, and cytotoxic properties. Embryonic toxicology testing done using zebrafish showed that toxicity increases with higher concentrations. This should be kept in mind while considering using this formula as a local delivery drug. Hence, melatonin-loaded neem and clove extract can be further formulated into various vehicles to be used against periodontal pathogens in periodontitis patients. However, animal or cell line studies are needed to ensure safety, heading into clinical application to periodontitis patients.
